# Pd/C‐Catalyzed Efficient and Selective Synthesis of Glycolic Acid from Ethylene Glycol

**DOI:** 10.1002/cssc.202501839

**Published:** 2025-10-23

**Authors:** Alban Schmoll, Yong‐Wang Huo, Sufang Shao, Xiao‐Feng Wu

**Affiliations:** ^1^ Dalian National Laboratory for Clean Energy Dalian Institute of Chemical Physics Chinese Academy of Sciences Dalian Liaoning 116023 China; ^2^ Leibniz‐Institut für Katalyse e. V. Albert‐Einstein‐Straße 29a 18059 Rostock Germany

**Keywords:** ethylene glycol, glycolic acid, green chemistry, oxidation, palladium on carbon

## Abstract

Palladium on carbon catalyst is used to develop an effective and selective system to produce potassium glycolate, a salt of glycolic acid, which is an interesting chemical with uses in many industries including the polymers and cosmetics industries. The mild conditions for this reaction allow to obtain a high yield of potassium glycolate, up to 92%, starting from ethylene glycol.

## Graphical abstract

A palladium on carbon catalyst system is developed for the synthesis of potassium glycolate from ethylene glycol, in up to 92% yield.

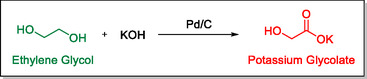

## 1 Introduction

**Scheme 1:**
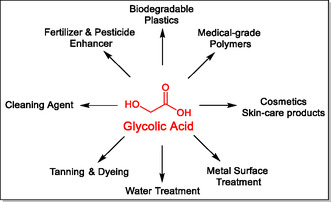
Glycolic acid: A versatile hemical with diverse applications.

Glycolic acid (GA) is an important chemical with applications in numerous fields (**Scheme** **1**). Considerable attention has been given to its use as a co‐monomer for bio‐degradable polymers and other sustainable materials for bioplastics.^[^ ^1^ ^]^ Such materials could replace conventional plastics commonly used in daily life including, plastic bags, food packaging, or disposable items such as coffee cups. In this context, GA‐based polymers have the potential to reduce the amount of non biodegradable plastic produced, thus reducing the environmental impact of our current consumption habits. This chemical has also been heavily investigated for biomedical purposes. Bio‐degradable materials based on GA, either as monomer or co‐monomer, display properties suiting for a drug delivery system or bone tissue engineering.^[^ ^2^, ^3^, ^4^ ^]^ Poly(lactic*‐*co‐ glycolic) acid (PLGA) polymers are extremely attractive due to their biocompatibility and variable degradation rate, which depends on the lactic acid/glycolic acid ratio. It has also been known for many years for its qualities in cosmetics for its efficiency as a peeling, antiaging, and acne treatment, with numerous studies supporting its efficiency.^[^ ^5^, ^6^, ^7^ ^]^ Today, it is present in a wide range of commercialized skin‐care products marketed globally. Additionally, GA has applications including tanning and dyeing in the textile industries, in the food industry as a preservative or in cleaning agents as well.

GA can be produced through different methods, including extraction from natural sources such as sugarcane, corn, or pineapple. However, chemical synthesis is more cost‐effective and does not come in competition with food or sugar production that which plants are usually grown for, which would make it particularly unattractive to develop a sustainable supply chain. Currently, it is mainly produced from fossil resources by either hydration of ethylene oxide or carbonylation of formaldehyde, which requires relatively harsh temperature and pressure conditions as well as the use of strong acids, which corrode reactors.^[^ ^8^, ^9^ ^]^ These drawbacks highlight the need for more sustainable methods and researchers have investigated new methods of synthesizing this compound. This is of particular importance to develop sustainable pathways using renewable resources for the synthesis of key chemicals, as the global climate crisis is urging us to reduce our consumption of fossil resources. In this context, a promising route to produce glycolic acid is to convert ethylene glycol (EG). EG can be a renewable chemical as it is obtainable from lignocellulosic biomass or cellulose. This could be an environmentally friendly alternative to current synthetic methods.^[^ ^8^ ^]^ It can also be produced from glycerol, a by‐product of the biodiesel industry obtained through the transesterification of triglycerides.^[^ ^9^ ^]^ Glycerol, being the main by‐product of this worldwide‐used process it is created in large amounts and, thus, its valorization is essential to ensure sustainability, especially in a context of environmental crisis. Using waste to obtain products such as EG that can then be transformed into a value‐added chemical used in cosmetics or polymers is both sustainable and attractive.^[^ ^10^, ^11^, ^12^ ^]^

Examples in the literature have already explored the transformation of EG to GA and different methods have been developed during the past years to achieve promising results for future applications in industry. Electrocatalysis methods were investigated using a variety of electrodes made of noble metals such as platinum, palladium, or gold, mainly used as alloys. One particularly attractive application is the use of EG for fuel cells, which can convert chemicals cleanly into electricity through redox reactions. Researchers, both in academics and industries are actively working on developing those systems that can output relatively clean energy, and EG is still being investigated.^[^ ^13^, ^14^, ^15^ ^]^ Other means to produce GA from EG were also reported. Some of them concern the use of oxidizing microorganisms. However, as illustrated by the results presented by Shimizu and coworkers, who were able to achieve up to 92% yield under 120 h, biological routes can be selective and effective, albeit slower.^[^ ^16^ ^]^ The isolation, cultivation, possible engineering, and use of these bacterial strains can also be challenging, but these results are very promising for possible future applications.

**Figure 1:**
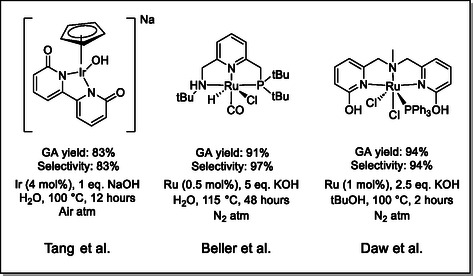
Homogeneous catalysts for the transformation of EG into GA.

Homogeneous catalysis also shows interesting results (**Figure** **1**). Daw et al. developed a switchable ruthenium‐based system to produce glycolic acid with yields up to 94% in 2 h at 100 °C under an inert atmosphere using 1 mol% of catalyst, 2.5 equivalents of potassium hydroxide, and *tert*‐butyl alcohol as (KOH) solvent.^[^ ^17^ ^]^ Those conditions, even though mild and enabling a high yield of glycolic acid, did not allow them to obtain selectively the targeted product. Beller and coworkers, thanks to a well‐defined ruthenium NNP pincer complex, were also able to achieve high yields using sodium hydroxide in water at a temperature of 115 °C. Using 0.5 mol% of their catalyst and 5 equivalents of base, they were able to obtain yields up to 91% after 48 h in a glovebox under an inert atmosphere of nitrogen.^[^ ^18^ ^]^ Another system has been developed by Tang and coworkers; using 4 mol% of a [Cp*Ir(bpyO)]OH^−^ homogeneous catalyst along with one equivalent of sodium hydroxide in water they were able to completely convert EG and obtain a yield of glycolic acid of 81.5% after 12 h at 100 °C.^[^ ^19^ ^]^ For this system, it is not required to work under an inert atmosphere; however, the selectivity toward the formation of glycolic acid is not excellent. These systems show the potential of EG to be transformed into glycolic acid in an efficient manner. Nevertheless, they all require the synthesis of complex ligands and their corresponding catalysts, while the conditions stay harsh for the majority. Moreover, most of the recent examples do not achieve selective transformation of EG and require operating under an inert atmosphere for them to be working. This underlines the need for a selective, efficient and more practical method.

In this study, we present a simple and efficient system for glycolic acid production using a commercially available palladium catalyst, palladium on charcoal. This method enables the selective formation of potassium glycolate in excellent yield selectively without the need for an inert atmosphere, offering a more practical and scalable alternative. Notably, Pd/C has several advantages, including high catalytic efficiency benefited from the large surface area of the activated carbon support, high stability, etc.^[^ ^20^, ^21^, ^22^ ^]^

## 2 Results and Discussion

For this study, our goal was to obtain glycolic acid from EG selectively and in high yield. We thus investigated different simple catalysts for this transformation. As shown in **Table** **1**, ruthenium‐based catalysts, despite being active in converting EG, were unselective toward the formation of our targeted product. We also tried to work with palladium on carbon (Pd/C), which showed high activity and proved to be much more selective toward the formation of GA, or more precisely, its salt potassium glycolate. The by‐products ruthenium catalysts were forming included potassium formate and other C1 products that were not detected by either ^1^ H or ^13^C NMR. Regarding already published results, it is possible that carbon dioxide and hydrogen were being formed in these reactions.^[^ ^17^, ^18^ ^]^ Water, *N*,*N*‐dimethylformamide (DMF), and *N*,*N*‐dimethylacetamide (DMA) were used as potential solvents for our system without leading to further improvement. Conversion put aside, water and DMA, however allowed to stay selective toward the formation of GA when using Pd/C. No desired product could be detected when Pd(OAc)_2_ was tested as the catalyst.

**Table 1:** Catalyst and solvent investigations.

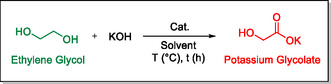					
Entrya)	Cat.	Solvent	Conv. EG [%]	Yield GA [%]b)	Sel. [%]
1	RuCl_3_	1,4‐dioxane	99	10	10
2	RuCl_2_(PPh_3_)_2_	1,4‐dioxane	80	27	34
3	Pd/C	1,4‐dioxane	75	75	99
4	Pd/C	H_2_O	8	8	99
5	Pd/C	DMF	12	6	50
6	Pd/C	DMA	17	17	99

a) Reaction conditions: EG (1 mmol), KOH (2.5 eq.), 2 mol% of metal catalyst, solvent (8 mL), 102 °C, 24 h.

b) Yields were obtained by ^1^ H NMR of the crude reaction mixture using 0.25 mmol of 1,6‐lutidine as internal standard. Pd/C (5 wt% loading (dry basis), matrix activated carbon support, powder).

After screening reaction temperatures with this catalyst, we determined that 110 °C is optimal for this reaction as it maximizes conversion while staying selective under mild conditions (**Table** **2**, entry 3). At this temperature, we were able to obtain an excellent potassium glycolate yield of 92%, making this system even more interesting. It is notable that lowering the temperature to 90 °C enables us to stay selective for the cost of making the conversion drop. Moreover, decreasing the temperature further led to a decline of conversion and selectivity. We thus focused our efforts on improving this system further at 110 °C. We then investigated the effects of different additives on our reaction conditions in the hope of further improving glycolic acid yields. First, we tried to add small amounts of water, as it might facilitate Cannizzaro reactions that might be happening as proposed in some articles.^[^ ^19^ ^]^ This did not affect selectivity but lowered conversion down to 70%, thus making water disadvantageous for our system (Table 2, entry 4). It is possible, as we are using wetted Pd/C catalyst, that the water content of the catalyst is already improving the reaction rate and further addition of water becomes detrimental. The water present in the catalyst could also reduce the performance of our system; however, as it is present to prevent the powder from being pyrophoric, it is preferable not to dry it. Using our standard reaction conditions at 110 °C, we also attempted to reduce the amount of base, KOH, to lower the overall process cost. It resulted in a significant drop in conversion and selectivity, highlighting that for our system, 2.5 equivalents of base are necessary using KOH (Table 2, entry 5). We also tried not to use any base at all to see if any reaction would occur and absolutely no conversion of the starting material was observed (Table 2, entry 7). Thus, Pd/C cannot convert EG in any way without the addition of base in the case of our system. We believe the base is important for the salt formation and drive the reaction to proceed effectively and selectively.

**Table 2:** Reaction temperature and additives investigations.

Entrya)	Cat.	T [°C]	Add.	Conv. EG [%]	Yield GA [%]b)	Sel. [%]
1	Pd/C	80	–	29	29	99
2	Pd/C	90	–	80	27	34
3	Pd/C	110	–	92	92(89)c)	99
4	Pd/C	110	25 μL H_2_O	70	70	99
5	Pd/C	110	2 eq. KOH	82	79	96
6	Pd/C	110	1.25 eq. KOH	75	63	84
7	Pd/C	110	no KOH	0	0	–
8	Pd/C	110	N_2_ atm.d)	87	87	99
9	Pd/C	110	O_2_ atm.e)	92	76	83

a) Reaction conditions: EG (1 mmol), KOH (2.5 eq.), 2 mol% of metal catalyst, 1,4‐dioxane (8 mL), T (°C), 24 h.

b) Yields were obtained by ^1^ H NMR of the crude reaction mixture using 0.25 mmol of 1,6‐lutidine as internal standard.

c) Isolated yield of benzyl glycolate after esterification of potassium glycolate with benzyl halide.

d) Liquid components were added in a N_2_ filled glovebox before closing the reactor.

e) Oxygen was bubbled in the reactor and it was closed so that most of the air was replaced.

**Scheme 2:**
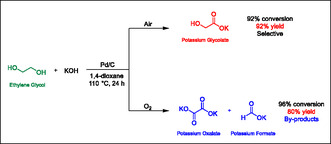
Reaction pathways and by‐product formation under air and oxygen.

Then we also examined the impact of the atmosphere on conversion. After using a nitrogen atmosphere for the reaction, it was determined that replacing air completely with N_2_ was detrimental to the conversion of EG, but not the selectivity toward GA. This result hints that molecular oxygen is playing a direct role concerning the conversion of EG leading to the formation of GA. Thus, we tried to charge our reactor with O_2_ to confirm this hypothesis and, hopefully, obtain higher yields (**Scheme** **2**). This resulted in the selectively to drop severely, mainly due to over‐oxidation (Table 2, entry 9). New by‐products, namely potassium oxalate and potassium formate, which were not obtained when working under an air atmosphere, were formed when bubbling O_2_.

It seems that, in the case of our system, O_2_ plays a role in the conversion of EG into GA, but that excess of it leads to selectivity issues and the formation of by‐products of over‐oxidation. Optimal conditions thus require working under air, which is convenient for potential applications, as it is not necessary to operate under an inert atmosphere. It would require in‐depth analysis to determine the exact role of this gas in our reaction, but its effect on the system is evident.

Further improvements to our conditions might be possible and could make this process viable on larger scales for industrial applications using renewable and sustainable EG as a starting material in the future.

## 3 Conclusion

This work reports an effective and convenient system for the transformation of EG, a readily available chemical that can be produced from renewable sources, into glycolic acid, which is heavily used for many applications going from different types of polymers to cosmetics. NMR yields up to 92% were obtained while being completely selective, using low loading of a simple commercial palladium catalyst under relatively mild conditions.

## Supporting Information

The authors have cited additional references within the Supporting Information.

## Conflict of Interest

The authors declare no conflict of interest.

## Supporting information

Supplementary Material

## Data Availability

The data that support the findings of this study are available in the supplementary material of this article.
