# Supplementary material for: Pd/C‐Catalyzed Efficient and Selective Synthesis of Glycolic Acid from Ethylene Glycol
Source: ChemSusChem. 2025 Oct 23;18(24):e202501839. doi: 10.1002/cssc.202501839 (PMC12703458; doi:10.1002/cssc.202501839)
Supplement: Supplementary file 1 — Supplementary Material [file CSSC-18-e202501839-s001.pdf]

Supporting information

1. General Experimental Information

All experiments were performed under air atmosphere unless specified. All reactions were performed in 15 mL heavy-wall reactor from Synthware. All chemicals purchased were used without further purification. Column chromatography was performed on silica gel (200-300 meshes) using a mixture of ethyl acetate and petroleum ether (2:8) as solvent. ^{1}H and ^{13}C NMR spectra were recorded at ambient temperature using a Bruker Avance III 400 MHz NMR (^{1}H, 400 MHz; ^{13}C {^{1}H}, 101 MHz). The chemical shift values of all compounds on ^{1}H NMR spectrum were reported with reference to water in D_2O (4.79 ppm).

2. General Procedure for Ethylene Glycol Conversion to Potassium Glycolate

In a heavy-wall glass reactor were placed catalyst (in mol%) and KOH (equivalent in respect to ethylene glycol) before adding solvent and finally ethylene glycol (1mmol). After all compounds were placed in the reaction tube, it was closed tightly and heated to the target reaction temperature for the mentioned reaction time. After a reaction was completed, the mixture was cooled down to room temperature before opening the reactor. Solvent was removed, residues in the reactor were cleaned with purified water and all were gathered into a round-bottom flask before evaporating 1,4-dioxane using a rotary evaporator. After 1,4-dioxane was removed, 1,6-lutidine (0.25 mmol, 0.25 eq.) was added to the mixture in water as internal standard before performing ^{1}H NMR or/and ^{13}C NMR. If evaporation to dryness, including water, distilled water was added to the round-bottom flask before adding internal standard. Conversion of ethylene glycol and yield of products were determined thanks to ^{1}H NMR data using the following calculations:

- Conversion of Ethylene Glycol (in %) = (1 mmol of EG – x mmol of EG remaining) / 1 mmol

- Yield (in %) of Potassium Glycolate = (x mmol Potassium Glycolate / 1 mmol of EG) x 100

- Selectivity (in %) of Potassium Glycolate = Conversion of Ethylene Glycol / Yield of Potassium Glycolate

3. NMR data

Peaks at 4.79 are from water, Peaks at 3.74-3.77 are from 1,4-dioxane

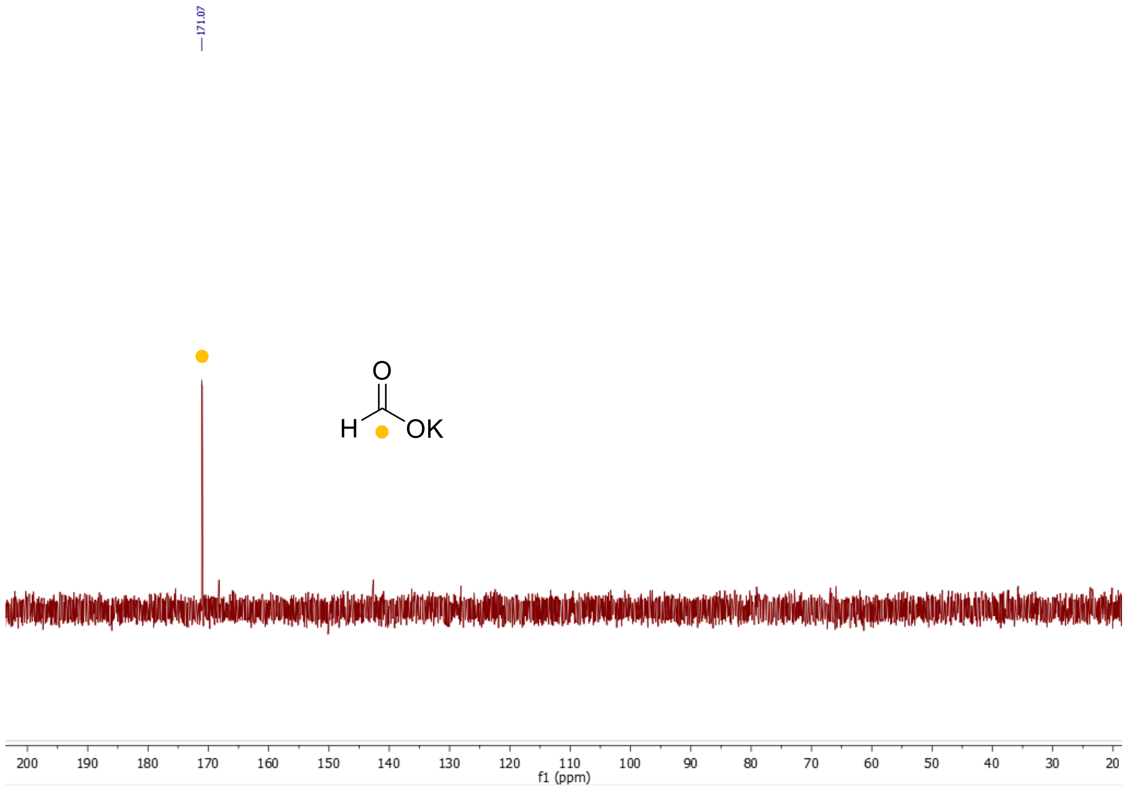

Figure 1: ^{13}C NMR spectrum in D_2O of potassium formate, synthesized by mixing potassium hydroxide and formic acid

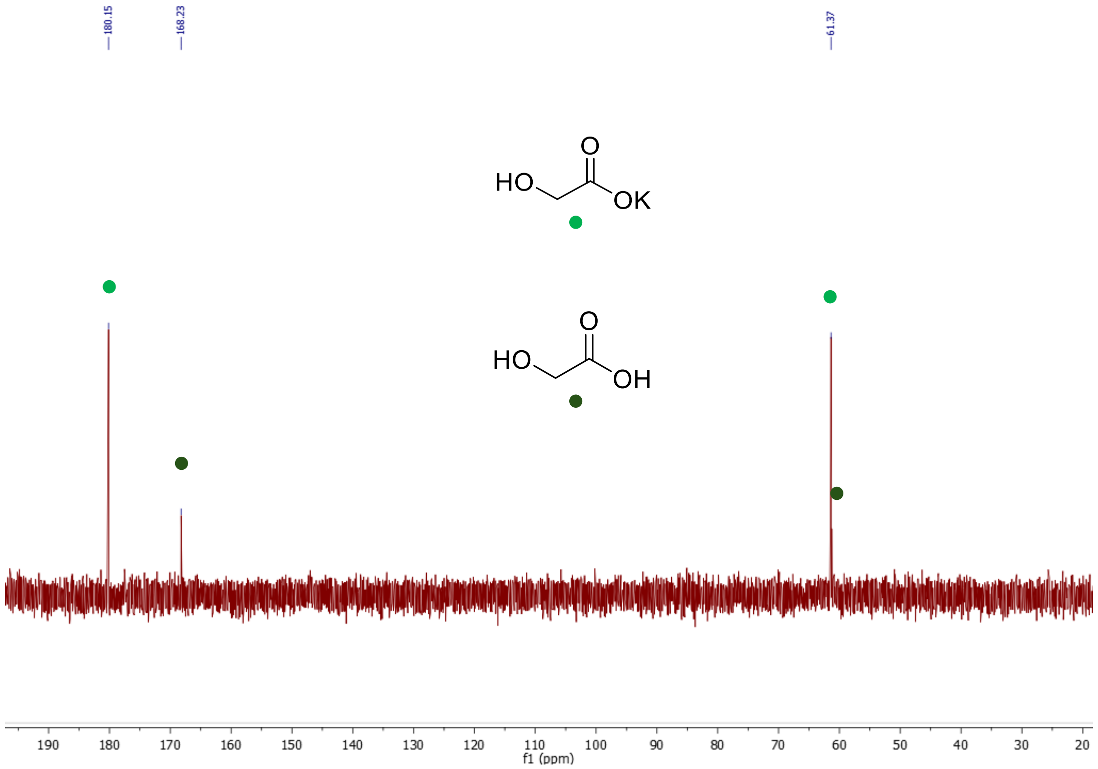

Figure 2: ^{13}C NMR spectrum in D_2O of potassium glycolate, synthesized by mixing potassium hydroxide and glycolic acid

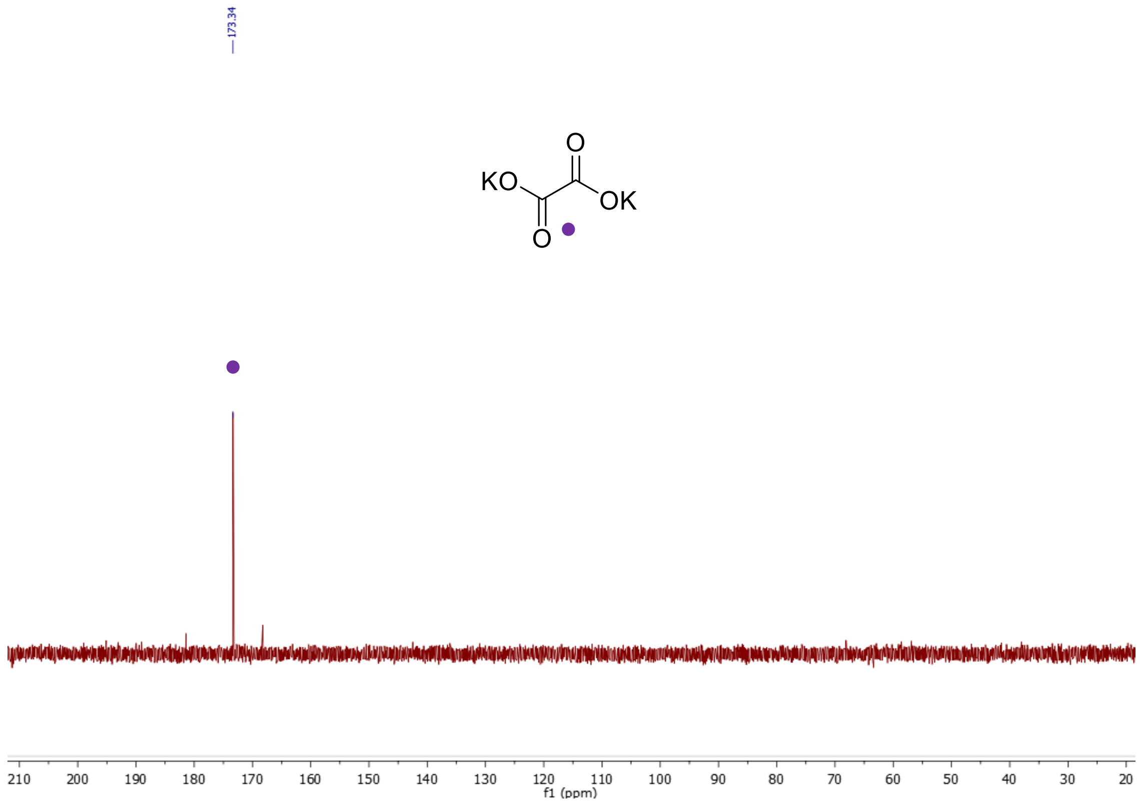

Figure 3: ^{13}C NMR spectrum in D_2O of potassium oxalate, synthesized by mixing potassium hydroxide and oxalic acid

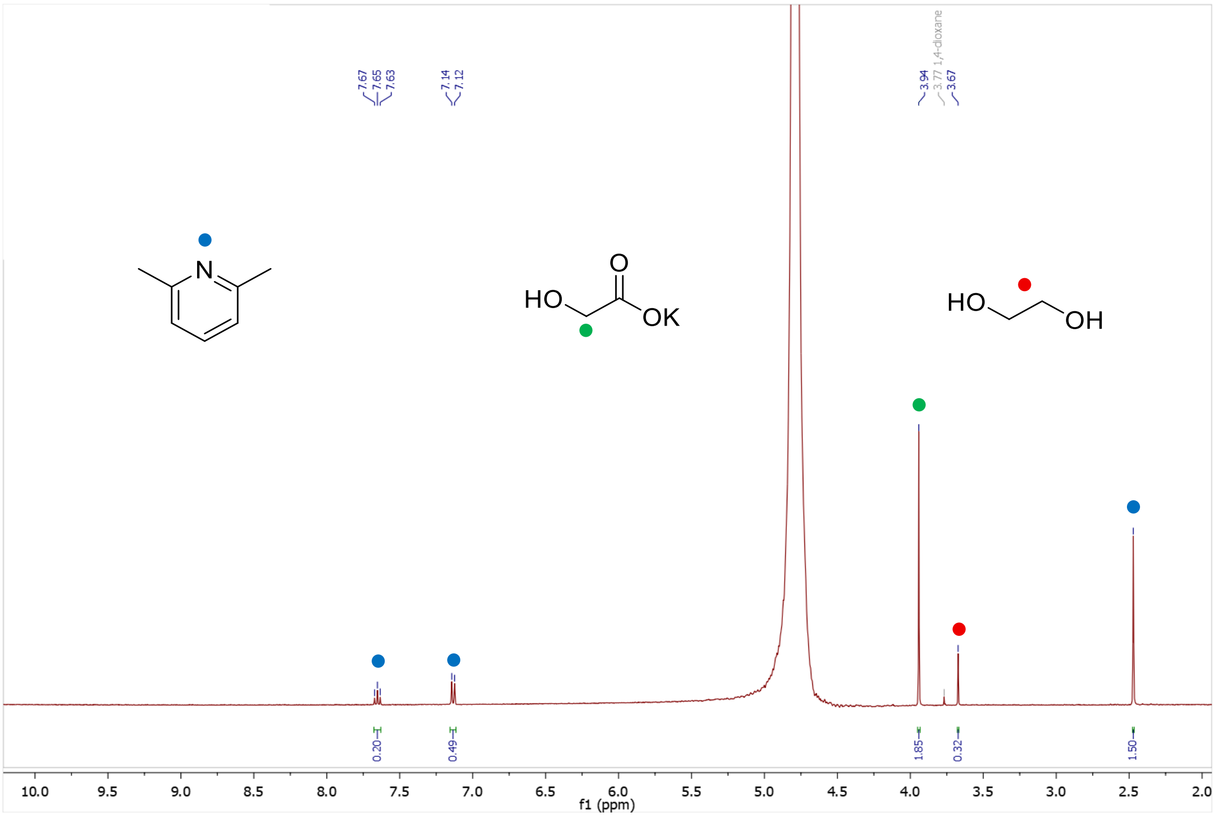

Figure 4: ^{1}H NMR spectrum in D_2O of the crude reaction mixture for dehydrogenation of ethylene glycol into potassium glycolate (Table 2, Entry 3)

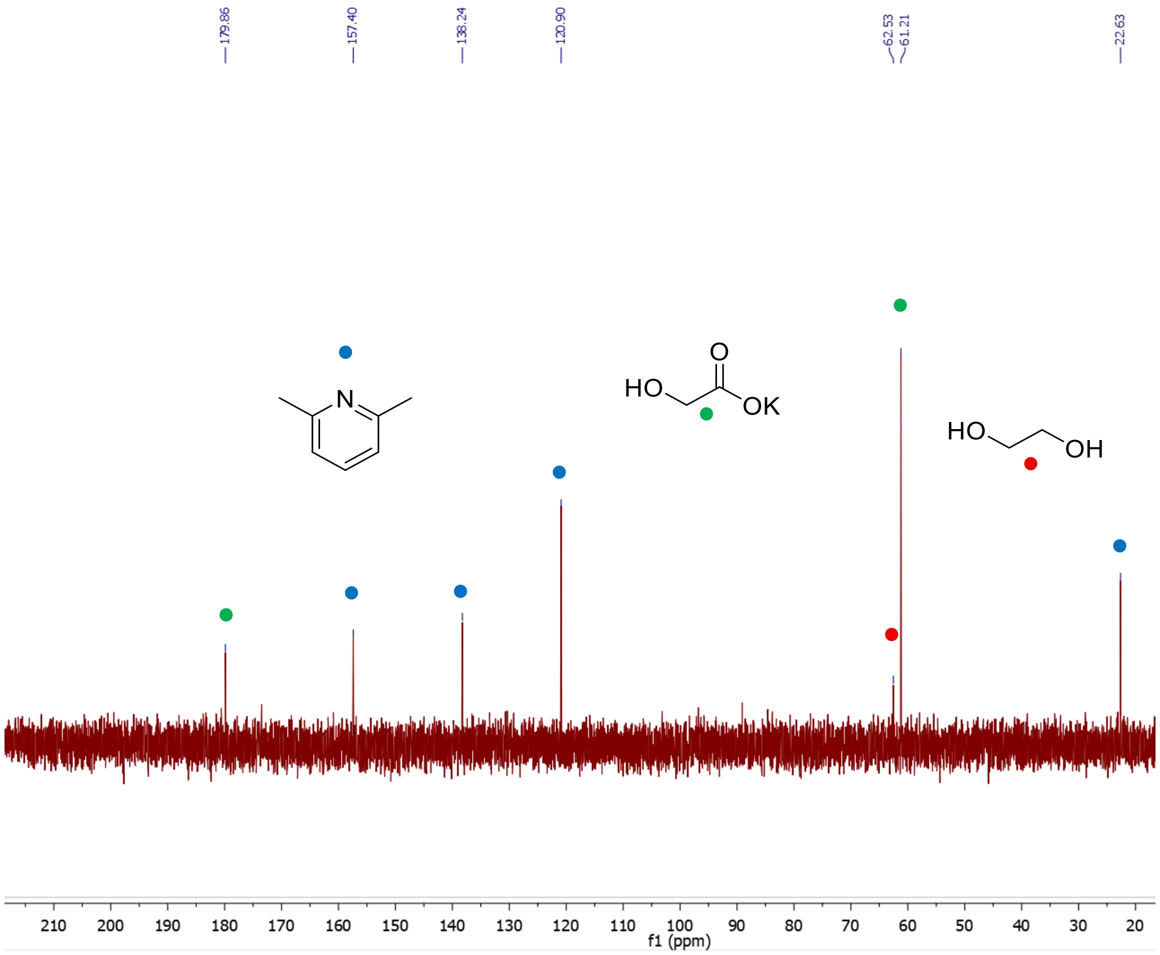

Figure 5: ^{13}C NMR spectrum in D_2O of the crude reaction mixture for conversion of ethylene glycol into potassium glycolate, Table 2, Entry 3

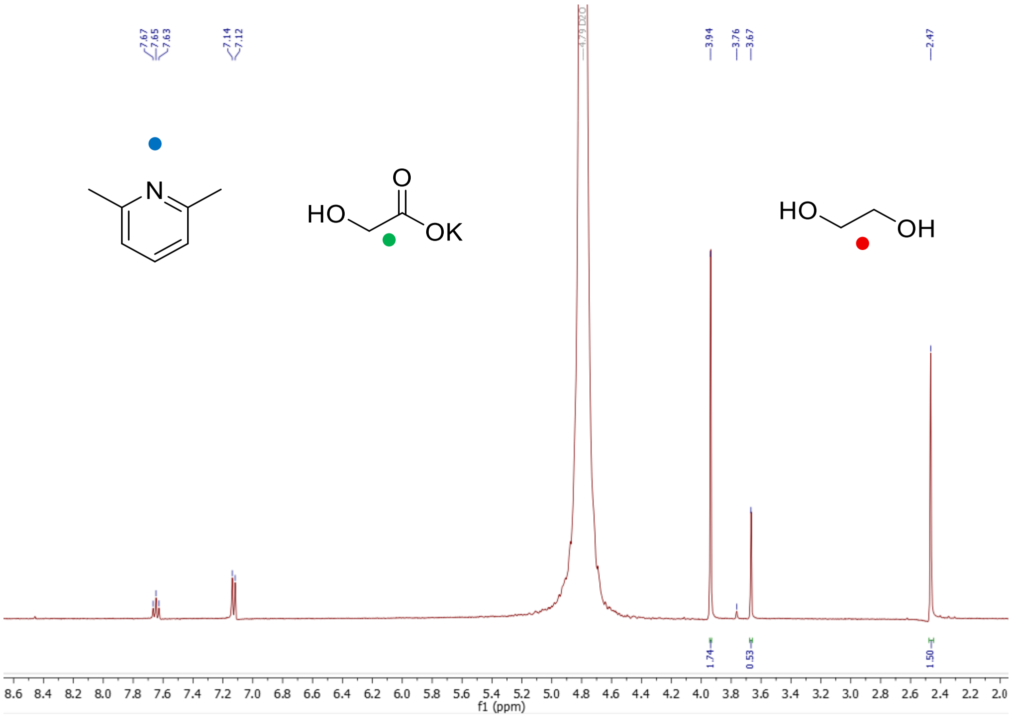

Figure 6: ^{1}H NMR spectrum in D_2O of the crude reaction mixture for conversion of ethylene glycol into potassium glycolate under N_2, Table 2, Entry 8

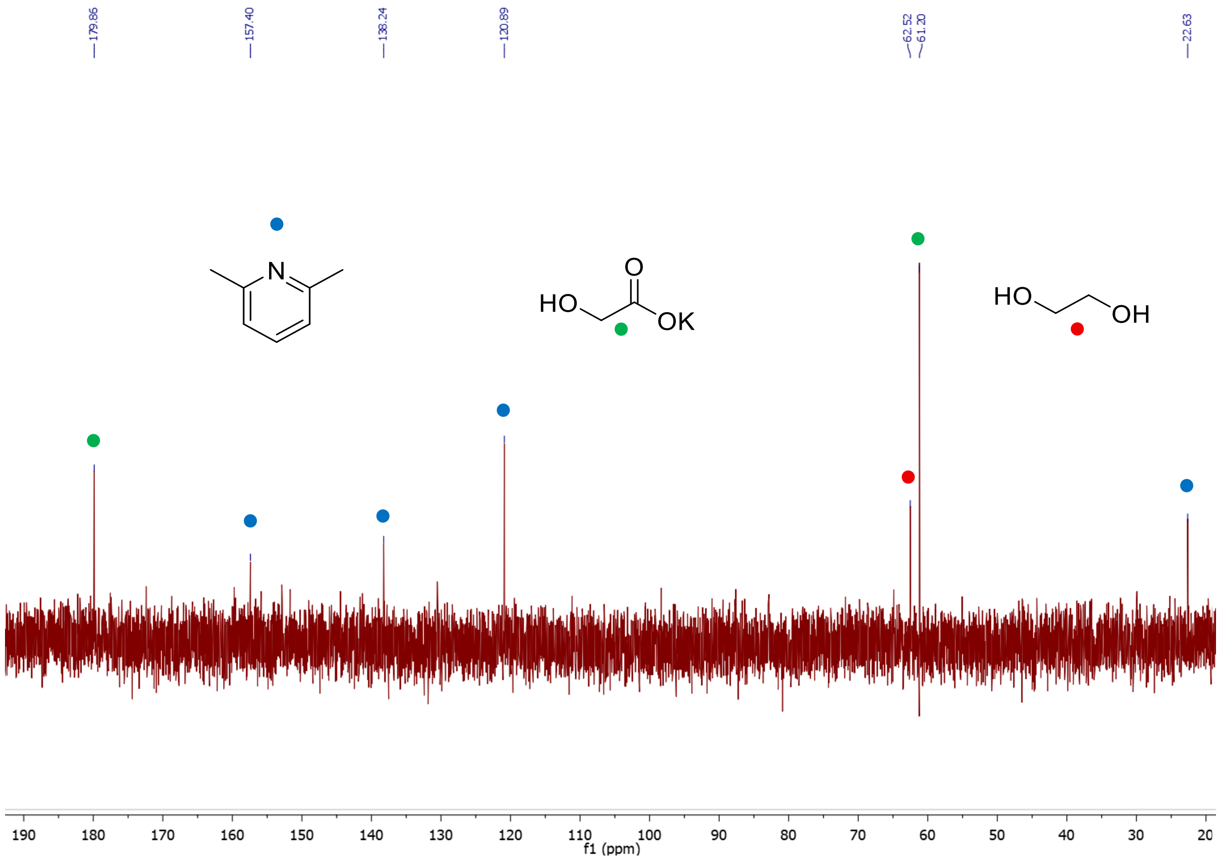

Figure 7: ^{13}C NMR spectrum in D_2O of the crude reaction mixture for conversion of ethylene glycol into potassium glycolate under N_2, Table 2, Entry 8

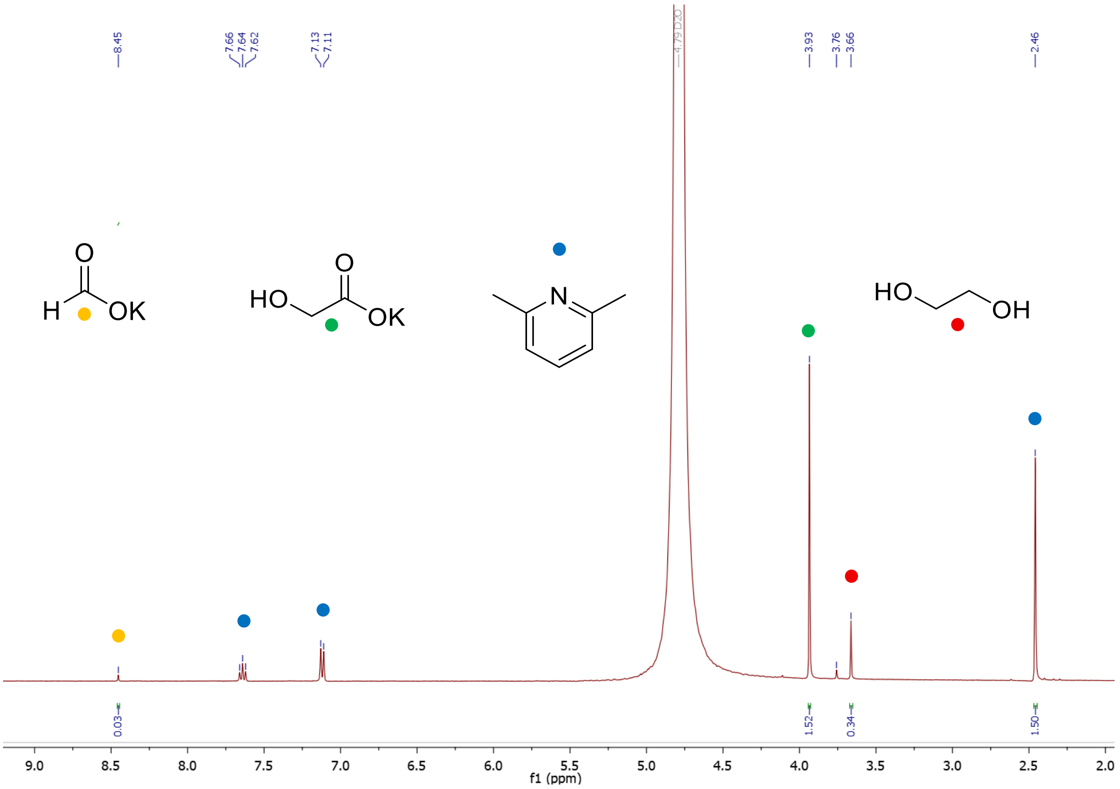

Figure 8: ^{1}H NMR spectrum in D_2O of the crude reaction mixture for conversion of ethylene glycol into potassium glycolate under O_2, Table 2, Entry 8

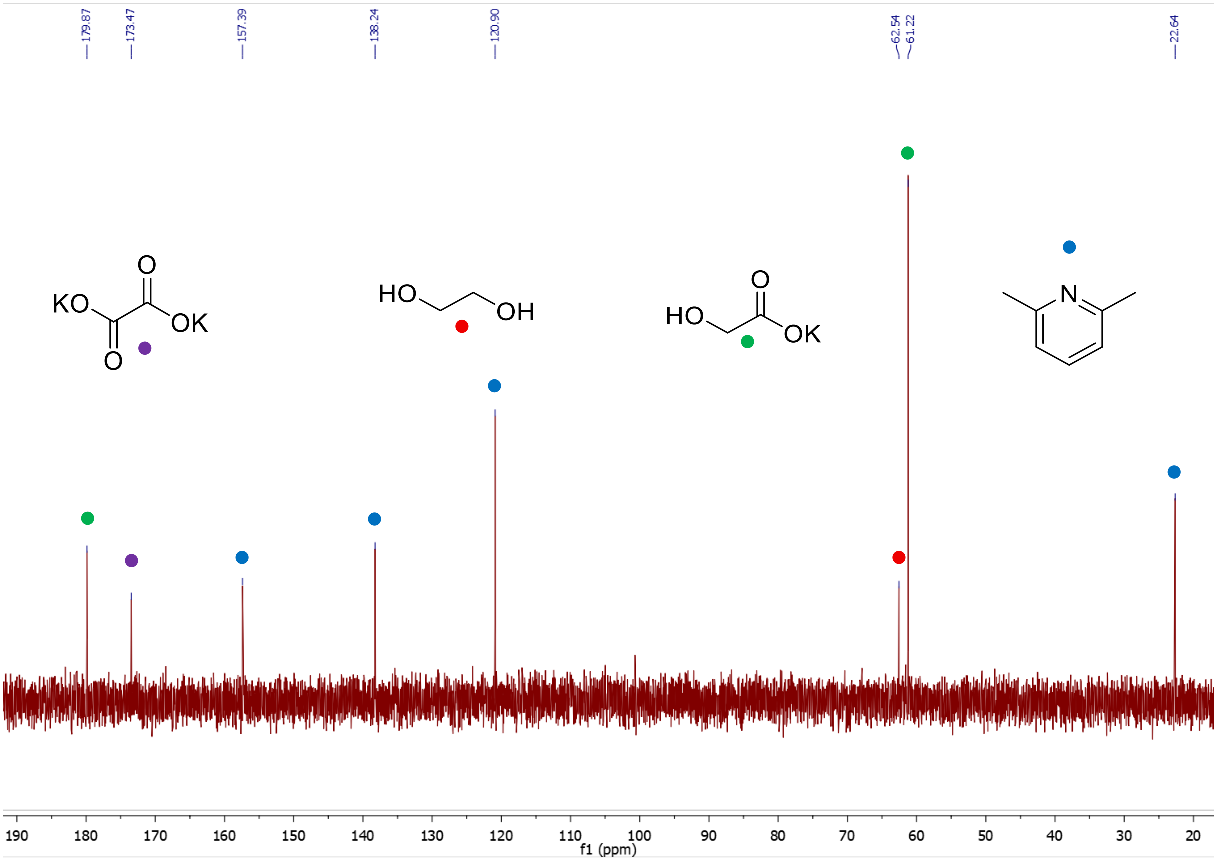

Figure 9: ^{13}C NMR spectrum in D_2O of the crude reaction mixture for conversion of ethylene glycol into potassium glycolate under O_2, Table 2, Entry 8
